# The Laymen's Influence in Public Medicine

**Published:** 1914-06-13

**Authors:** 


### The Hospital

The Workers' Newspaper of Administrative Medicine and Institutional Life, Administration, National Insurance and Health.

No. 1459, Vol. LVI. SATURDAY, JUNE 13, 1914. PRICE ONE PENNY.

### THE LAYMEN'S INFLUENCE IN PUBLIC MEDICINE.

"The mistake freely made by election committees of laying stress upon the administrative side" in appointments in some branches of medical work, which was the subject of remark in the leading article of The Hospital for June 6, brings prominently forward two very important questions—namely, the influence of bodies of laymen in administrative and preventive medicine, and the absorption of the energies of medical men by office-work, possibly to the detriment of clinical work and research.

We live in the era of the birth of administrative medicine, and the characteristics of our generation will, in all probability, be accentuated in the phases which will follow in the evolutionary "progress" of future generations. Medicine cannot be excluded and cannot stand aside; the spirit of communism will mould it, as it will mould all else, in those social developments which are slowly but surely proceeding. Excluding cases of favouritism in the making of appointments, and allowing for the natural tendency of bodies of laymen to appoint medical men with a local reputation, such bodies must be generally credited with the desire to appoint the best man amongst the applicants for an appointment. It is, however, inevitable that in many instances it must be administrative qualities which attract and impress the members of an appointing authority. These members can seldom assess the value of purely medical work; they know that they may be misled by claims, possibly exaggerated, to exceptional medical experience or research; they become confused amongst a mass of diplomas and effusive testimonials. Is it to be wondered at that they fall back upon an attempt to find out what a candidate has *done*, if not within the range of their personal knowledge of him and of his work, at least within that of their general knowledge of affairs? In such a way may arise the importance attaching to administrative experience to those seeking public medical appointments. It is not only in connection with the appointment of tuberculosis medical officers that the "mistakes" referred to have been made.

In the work of a medical man holding a public appointment there is again a direct and very important influence due to the control of the lay authority, and depending upon the quality, aims, and breadth of view of that authority. Medical men holding appointments have in only too many instances no security of tenure, no prospect of pension, and but little hope of improved position; they are dependent upon the authority under which they work and which has large powers, collectively and often individually, to make or mar the officer's reputation and, if so minded, to harass him in the performance of his duties. Here again it is administrative work which is likely to count and of which the effects are obvious; a popular article in the local paper makes more impression than a statistical inquiry concerning the local incidence of an obscure disease. Some of those which may be called minor public bodies now employ and control a large staff. Of the work of most of those employed the members can judge, and with it individual members even occasionally interfere. When one medical officer is upon the staff there may be a tendency to judge his work by the same standard, to seek some tangible evidence that he is kept busy, and to make too much of the excuse that it is a duty to see that value is being obtained for public money expended. There may even be shown a jealousy of the independence resulting from his professional knowledge and his isolation. The members of such authorities have generally but crude and, at the best, utilitarian ideas of preventive medicine. Enough has been said to show that there are existing influences which may discourage or even stifle medical ardour.

It may be stated, without fear of contradiction, that the time and energies of most of those occupying public medical appointments are nowadays largely taken up by work not purely medical. This applies especially to those higher posts which are often actually spoken of as administrative. As the scope of an appointment grows, as the number of assistant medical and other officers increases, so the office work becomes more complicated and engrossing, and the chief medical officer becomes more of an adviser, supervisor, and organiser; so that it may almost be said that, in administrative medicine, the opportunity for personal or original medical work diminishes in proportion with increase of salary. This condition applies more or less to the principal medical officers of all our great public bodies and of all our great public institutions. Medical men are appointed to important administrative posts because only highly-trained and experienced medical men are competent to hold them, and the "office-work" is skilled and specialised office-work. The medical officer of health and the tuberculosis medical officer have, although the duties of the latter are "very largely clinical," much office work to do and to control; so will the medical man appointed under the Act dealing with the feeble-minded. Most "public health" medical officers have, then, duties which debar them from extended research and are provided with no means for expensive experiment.

Is undue importance attaching to the office-work aspect of public medical work? It is to be feared that the tendency is in that direction. It is for medical men, both individually and through such societies as the Society of Medical Officers of Health, to prevent the reference-numbered type-written foolscap letter from becoming a fetish or the card-index an obsession.

